# Building on existing tools to improve chronic disease prevention and screening in public health: a cluster randomized trial

**DOI:** 10.1186/s12889-021-11452-x

**Published:** 2021-08-03

**Authors:** A. K. Lofters, M. A. O’Brien, R. Sutradhar, A. D. Pinto, N. N. Baxter, P. Donnelly, R. Elliott, R. H. Glazier, J. Huizinga, R. Kyle, D. M. Manca, M. A. Pietrusiak, L. Rabeneck, B. Riordan, P. Selby, K. Sivayoganathan, C. Snider, N. Sopcak, K. Thorpe, J. Tinmouth, B. Wall, F. Zuo, E. Grunfeld, L. Paszat

**Affiliations:** 1 Department of Family & Community Medicine, University of Toronto, Toronto, Canada; 2 Women’s College Hospital Research Institute, Toronto, Canada; 3 Peter Gilgan Centre for Women’s Cancers, Women’s College Hospital, Toronto, Canada; 4 Ontario Health (Cancer Care Ontario), Toronto, Canada; 5 ICES, Toronto, Canada; 6 Institute of Health Policy, Management and Evaluation, University of Toronto, Toronto, Ontario Canada; 7 MAP Centre for Urban Health Solutions, St. Michael’s Hospital, Toronto, Canada; 8 Department of Family and Community Medicine, St. Michael’s Hospital, Toronto, Canada; 9 Division of Biostatistics, Dalla Lana School of Public Health, University of Toronto, Toronto, Ontario Canada; 10 Dalla Lana School of Public Health, University of Toronto, Toronto, Canada; 11 University of St. Andrews, Scotland, UK; 12 Durham Region Health Department, Whitby, Canada; 13 Melbourne School of Global and Population Health, University of Melbourne, Melbourne, Australia; 14 Department of Family Medicine, University of Alberta, Edmonton, Canada; 15 Centre for Addiction and Mental Health, Toronto, Canada; 16 Applied Health Research Centre, St. Michael’s Hospital, Toronto, Canada; 17 Sunnybrook Health Sciences Centre, Toronto, Canada; 18 Ontario Institute for Cancer Research, Toronto, Canada

## Abstract

**Background:**

The BETTER (Building on Existing Tools to Improve Chronic Disease Prevention and Screening in Primary Care) intervention was designed to integrate the approach to chronic disease prevention and screening in primary care and demonstrated effective in a previous randomized trial.

**Methods:**

We tested the effectiveness of the BETTER HEALTH intervention, a public health adaptation of BETTER, at improving participation in chronic disease prevention and screening actions for residents of low-income neighbourhoods in a cluster randomized trial, with ten low-income neighbourhoods in Durham Region Ontario randomized to immediate intervention vs. wait-list. The unit of analysis was the individual, and eligible participants were adults age 40–64 years residing in the neighbourhoods. Public health nurses trained as “prevention practitioners” held one prevention-focused visit with each participant. They provided participants with a tailored prevention prescription and supported them to set health-related goals. The primary outcome was a composite index: the number of evidence-based actions achieved at six months as a proportion of those for which participants were eligible at baseline.

**Results:**

Of 126 participants (60 in immediate arm; 66 in wait-list arm), 125 were included in analyses (1 participant withdrew consent). In both arms, participants were eligible for a mean of 8.6 actions at baseline. At follow-up, participants in the immediate intervention arm met 64.5% of actions for which they were eligible versus 42.1% in the wait-list arm (rate ratio 1.53 [95% confidence interval 1.22–1.84]).

**Conclusion:**

Public health nurses using the BETTER HEALTH intervention led to a higher proportion of identified evidence-based prevention and screening actions achieved at six months for people living with socioeconomic disadvantage.

**Trial registration:**

NCT03052959, registered February 10, 2017.

## Introduction

In Ontario (Canada’s most populous province), adults with the lowest socioeconomic status are less likely to participate in preventive actions for cancers and chronic diseases. Specifically, they are less likely to be non-smokers, physically active, and consuming adequate fruits and vegetables than those with the highest socioeconomic status [1]. Similarly, screening for cancers, for cardiovascular disease and for diabetes, all evidence-based actions with well-established evidence for decreasing morbidity and mortality, are lowest in Ontario in low-income neighbourhoods, particularly among residents without a regular primary care physician [2]. These disparities in the uptake of prevention and screening exist despite a universal health care system, where access to primary care services including cancer screening and preventive health checks are free to Ontario residents. Canadian and Ontario studies have found the incidence and prevalence of lung cancer, cervical cancer and diabetes is highest among residents of low-income neighbourhoods [3–5].

A potential barrier to surmounting these income-related disparities is that prevention and screening activities are often promoted as individual discrete activities as opposed to an integrated set of evidence-based chronic disease preventive and screening actions [6]. People living with low income may have challenges in accessing and attending appointments and may experience competing priorities at healthcare visits, making it more difficult for these individual activities to be adequately addressed [7]. The original BETTER (Building on Existing Tools To Improve Chronic Disease Prevention and Screening in Primary Care) intervention was designed to integrate the approach to, and thus optimize participation in, prevention and screening actions [6]. The BETTER intervention was targeted at primary care patients aged 40 to 65 years and involved assessing a person’s risk factors and current participation in evidence-based prevention and screening actions, and using principles of motivational interviewing and brief action planning to support them to set their own goals. The intervention was administered by a “prevention practitioner” and consisted of a dedicated prevention-focused visit. The prevention practitioner was an existing non-physician health professional already situated within primary care practices (e.g. nurses, dieticians) and specifically trained to be a prevention practitioner. The BETTER intervention was evaluated in a cluster-randomized trial, and found a 32.5% increase in the number of prevention and screening actions that were met by patients in the intervention group versus the control group [6].

However, participants in the original BETTER trial were by definition well-connected to primary care and were of relatively high socioeconomic status (approximately half had a household income of $100,000 or more). In this study, we aimed to adapt BETTER (the adaptation is hereafter referred to as “BETTER HEALTH”) for those who may be the most marginalized by the healthcare system and most in need of prevention-focussed initiatives i.e. those living with socioeconomic disadvantage and who do not necessarily have connections to primary care. We designed BETTER HEALTH to be delivered through local public health instead of primary care, and to engage people through community organizations and direct communication strategies. Thus, our objective was to test the effectiveness of the BETTER HEALTH intervention at improving participation in chronic disease prevention and screening actions for residents of low-income neighbourhoods six months after informed consent compared to residents of low-income neighbourhoods randomized to wait-list control.

## Methods

### Trial design

The full protocol of this cluster non-blinded randomized trial with a 1:1 allocation ratio has previously been published [8]. There were no significant changes to methods after trial commencement. Ethics approval was provided by the research ethics boards of Sunnybrook Health Sciences Centre, Unity Health Toronto, the University of Toronto and the Durham Region Health Department.

### Study setting and participating clusters

Neighborhood clusters, or dissemination areas, were the unit of randomization. A dissemination area is a small, relatively stable geographic unit defined by the Canada Census. Durham Region is an area of Ontario east of Toronto with an estimated 2019 population of 699,460 people [9]. Twenty-two neighborhoods, or clusters, in Durham were eligible for the study based on average household income and cancer screening rates i.e. they were in the lowest income quintile based on Census data and had the lowest levels of cancer screening participation (less than 60% for cervical cancer screening, less than 55% for breast cancer screening, and less than 50% for colorectal cancer screening).

Ontario has 35 public health units, which serve to administer health promotion and disease prevention programs to the public in their regions on topics such as healthy lifestyles and communicable disease control [10]. Public health nurses in Ontario conduct a broad range of activities including providing clinical services (e.g. sexual health, immunizations), conducting home visits, providing health education, participating in outbreak and infectious disease management, and supporting community members to address the social determinants of health (SDOH) including connecting them with relevant local resources. Staff from the local public health unit, Durham Region Health Department, were eager to be active partners and co-investigators in the study and agreed that public health nurses would be well suited for the role of prevention practitioner. As well, our identified neighborhoods aligned with neighborhoods that had already been designated as a priority by the public health unit.

Three of the original twenty-two neighborhoods were excluded from randomization based on the public health unit assessment that these neighborhoods were atypical of the region (consisting of large apartment buildings), and then sixteen neighborhoods were randomly selected from those nineteen for inclusion in the sampling frame. We determined that we could reach the desired sample size from ten neighborhoods, but randomly selected sixteen in case we were not able to meet sample size with the first ten neighborhoods. All sixteen were randomly allocated to one of the study arms before participants were recruited. Co-author RS randomly selected the neighborhoods using the “sample()” function contained in the statistical software package ‘R’ [11]. A random number generator in R was then used to randomly allocate neighborhoods to each arm: immediate BETTER HEALTH versus six-month waitlist, and the allocation was then shared with the rest of the study team. As the code in R was written and implemented simultaneously, no allocation concealment was necessary.

### Study participants and recruitment

Individual participants were the unit of analysis for outcome assessment. Men and women aged 40 to 64 years of age who resided in one of the ten study neighborhoods were eligible to participate in the trial if they spoke English and were able to provide written informed consent. People who had participated in focus groups or interviews about the adaptation of BETTER HEALTH [8] were ineligible. Only one person per household was eligible. Participants were informed about the study using a variety of recruitment strategies, including posters, booths and presentations at local events, canvassing, and mailed flyers by the study research coordinator and/or prevention practitioners [12–14]. Interested individuals were invited to contact the study research coordinator by telephone to inquire further about participation.

### Data collection

All baseline and outcome data were determined by self-report in both arms, unlike the original BETTER where data came from both self-report and the medical record [6], as we had no access to participant records. We accordingly adapted baseline and outcome surveys from the original BETTER surveys. The survey queried current and past health status, medical history, family history, and sociodemographic characteristics. The study research coordinator verbally administered all surveys to participants in both arms to address concerns around literacy levels of participants, ensure clear understanding of all questions and help to build rapport. She administered surveys in a variety of settings: homes, the public health unit office, community centres, and other safe venues identified by the participants and research coordinator. The research coordinator recorded responses on paper surveys and transferred them afterward to a computer database on a laptop with privacy/confidentiality controls that were compliant with the requirements of the Office of the Privacy Commissioner of Ontario and the relevant research ethics boards. After baseline survey completion, participants in both arms received transit tickets (if travel was required), educational materials from the public health unit, and a $25 (Canadian) grocery gift card to compensate them for their time.

### Intervention

We trained three public health nurses to be prevention practitioners, with two prevention practitioners in the role at any given time. They underwent the usual two-day training curriculum on the BETTER intervention that included evidence-based guidelines, principles of motivational interviewing, brief action planning and shared decision-making in the context of small group discussions, cases, and role-playing [6, 15, 16]. They were trained on how to review a baseline survey to create a “prevention prescription” for participants and how to review this information with participants. They were also trained on how to support participants to set one to three specific, measurable, attainable, realistic and timely health-related goals, such as around smoking, diet, or physical activity [17]. A maximum of three goals was chosen to ensure that participants would not be overwhelmed and to increase the chances of success with achieving their goals. Goals were determined by the participant, not by the prevention practitioner, and were chosen based on what the participant felt most motivated to achieve and able to begin working on in the next week.

After reviewing participant responses from the baseline survey, prevention practitioners held an approximately 1–1.5 h prevention-focused visit with each participant. At the visit, the prevention practitioner reviewed their assessment of the participant’s overall health, provided the prevention prescription that documented current health status, evidence-based targets and relevant referrals suggested or made, and supported the participant to set their goals using brief action planning [16]. Upon getting a sense of their overall health status from the prevention prescription, participants then chose the goals that were most important and feasible to address. During the process of goal-setting, the prevention practitioners helped participants identify barriers to goals and discussed ways to overcome these barriers, including those related to the SDOH. Prevention visits occurred in mutually convenient and safe locations, such as the offices of the public health unit, community centres, participants’ homes, or other locations jointly agreed upon by the participant and the prevention practitioner. At the prevention visit, if the patient was eligible based on history and agreed, prevention practitioners measured height, weight, waist circumference and blood pressure using portable equipment. For the first four visits of the study, the two prevention practitioners reviewed together the baseline survey responses and the resulting prevention prescriptions for fidelity.

Prevention practitioners conducted a dedicated prevention visit with study participants in the intervention arm shortly after baseline data collection and with participants in the control arm after six-month outcome data collection.

Participants in the control arm completed the baseline survey and six-month survey, and were offered a visit with a prevention practitioner if they wished after the second survey was complete.

### Outcome measure

The primary outcome measure was adapted from that used in the original BETTER trial and was a composite index: the number of evidence-based prevention and screening actions achieved at six months as a proportion of those for which participants were eligible at baseline, measured at the individual level by self-report. The list of actions was adapted from the original 28 actions used in the BETTER trial, excluding nine actions that could not reasonably be determined by self-report (e.g. improvement in Framingham score) [18]. As a function of baseline characteristics, each individual was deemed eligible or not for each of nineteen evidence-based actions (for example, only current smokers were eligible for smoking cessation counselling). At the six-month follow-up, each participant was re-evaluated and the number of eligible actions that had been met was enumerated. If outcome data were missing, the action was considered as not met. Please see Appendix for details on the actions.

### Statistical analysis

We had a recruitment goal of 60 individuals age 40–64 years in each arm to allow us to detect an absolute mean difference of 30% or greater in the composite index (reflecting an increase in the proportion of actions met in the intervention arm versus the control arm. We chose 30% as the first BETTER trial saw a 30% improvement [6] . The sample size calculation was based on 80% power, 5% type I error rate, a standard deviation of 0.3, and an intra-cluster correlation coefficient of rho = 0.237 [8].

We calculated descriptive statistics of baseline characteristics for participants in both arms and used standardized mean differences (SMDs) to assess balance in the distributions of characteristics between arms. We determined the mean number of eligible actions at baseline, mean number of met actions at six-month follow-up, and the composite index for the study population overall as well as each study arm. For each individual action, we also determined the percentage of eligible individuals who met that action at follow-up. We determined the crude rate ratio (without accounting for clustering) for the intervention group compared to the wait-list group. To obtain an estimated rate ratio that accounted for the clustered design, we then developed a mixed-Poisson regression model that included a cluster-specific random effects term to account for correlation arising from individuals within the same cluster. The natural logarithm of the denominator (number of eligible items at baseline) was incorporated into the model as an offset term. The dependent variable was the number of met items at follow-up, and the exposure was the study arm. We also re-ran the model adjusting for household income, as we hypothesized that it was the most direct marker of the social determinants of health and may be associated with our outcome.

### Role of funding source

This study was funded by the Canadian Institutes of Health Research and the Canadian Cancer Society Research Institute. The funders had no role in the study design, conduct or reporting.

## Results

**Fig. 1:**
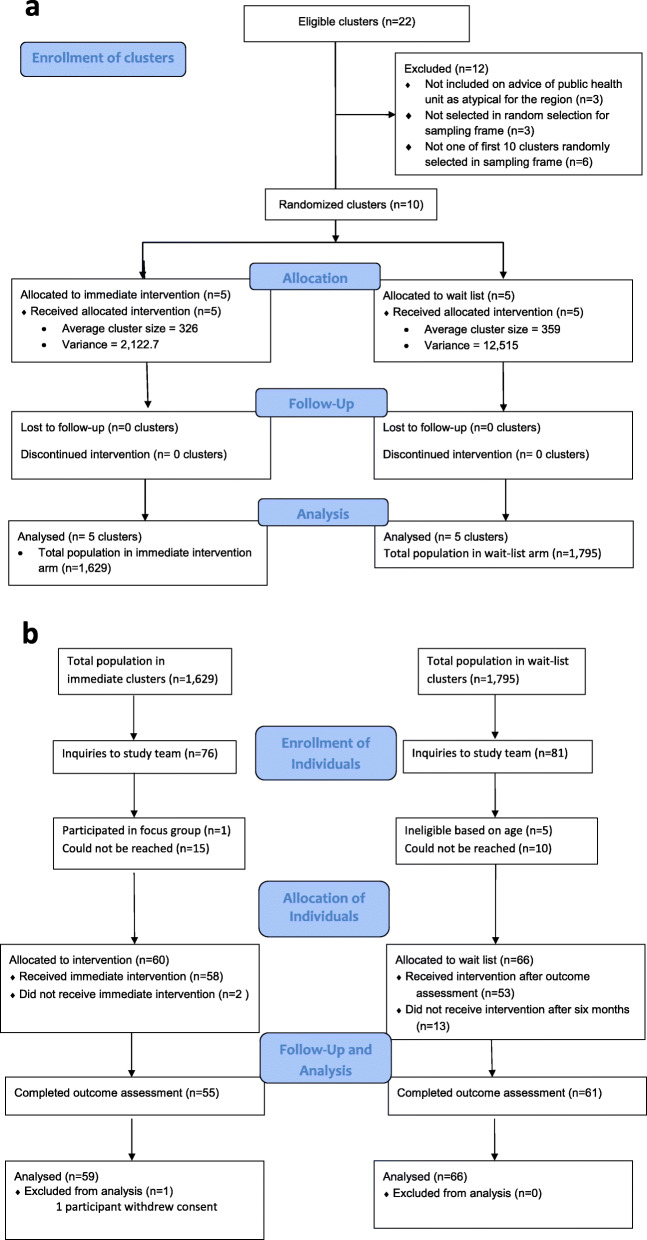
**A**. CONSORT Flow Diagram for Clusters (Neighborhoods). **B**. CONSORT 2010 Flow Diagram for Individuals

The CONSORT diagram for cluster allocation is shown in Fig. 1A and for individual outcome assessment in Fig. 1B. A total of 216 people made inquiries to the study team about participation. Of those, 59 people did not live in the clusters or could not be reached afterward to confirm their residence. Of the remaining 157 people who made inquiries, 76 lived in intervention neighborhoods and 81 lived in wait-list neighborhoods (Fig. 1B). A total of 25 people could not be reached after their initial inquiry, and six others were excluded due to age or having participated in a focus group. Sixty people in the immediate intervention arm and 66 in the wait-list arm consented to participate, were allocated to a study arm based on their neighborhood, and completed the baseline assessment. Five people in the immediate intervention arm and five people in the wait-list arm did not complete the outcome assessment. One participant in the intervention arm was not included in analyses due to withdrawal of consent. Trial recruitment started on October 2, 2017 and January 28, 2020 was the last date of data collection.

Table 1 presents baseline participant characteristics. Fewer participants in the intervention arm identified as male (35.6% vs. 48.5%, SMD = 0.26). Most participants were White. On average, participants’ body mass index (BMI) was over the threshold of 25 for overweight [19], with the wait-list participants having an average BMI of 30.8, exceeding the threshold of 30 for obesity [19]. Eleven participants overall (7 in immediate arm, 4 in wait-list arm) reported a household income of $60,000 or more, and 28 participants had not completed high school. Income, education and employment status differed between the two arms (SMD = 0.26, 0.56 and 0.51 respectively). More than one-third of participants reported being unable to work because of sickness or disability (32.2% in immediate intervention arm vs. 45.5% in wait-list arm). Most participants were neither married nor in a common-law relationship.

**Table 1:** Demographic characteristics of participants

	Level	Immediate	Wait-list	Missing
n		59	66	
Age (years)		54.5 (6.4)	53.2 (6.6)	0
Sex	Male	21 (35.6%)	32 (48.5%)	0
	Female	38 (64.4%)	34 (51.5%)	
Ethnicity	Caucasian/White	50 (84.7%)	52 (78.8%)	0
	Other	9 (15.3%)	14 (22.2%)	
Body Mass Index		28.7 (8.1)	30.8 (8.1)	4 (3.2%)
Household Income	Less than $10,000	16 (27.1%)	15 (23.1%)	1 (0.8%)
	$10,000 to $19,999	23 (39.0%)	32 (49.2%)	
	$20,000 to $39,999	9 (15.3%)	10 (15.4%)	
	$40,000 to $59,999	4 (6.8%)	4 (6.2%)	
	$60,000 or more	7 (11.9%)	4 (6.2%)	
Education	Elementary school or less	2 (3.4%)	5 (7.6%)	0
	Some high school	13 (22.0%)	8 (12.1%)	
	Completed high school	16 (27.1%)	21 (31.8%)	
	Some community college or technical school	4 (6.8%)	11 (16.7%)	
	Completed college or technical school	16 (27.1%)	14 (21.2%)	
	Some university	0 (0%)	2 (3.0%)	
	Completed bachelor’s degree	6 (10.2%)	4 (6.1%)	
	Graduate or professional degree	2 (3.4%)	1 (1.5%)	
Employment	Employed full-time	8 (13.6%)	5 (7.6%)	0
	Employed part-time	7 (11.9%)	11 (16.7%)	
	Unable to work because of sickness/disability	19 (32.3%)	30 (45.5%)	
	Looking after home/family	2 (3.4%)	2 (3.0%)	
	Student	0 (0%)	2 (3.0%)	
	Retired	6 (10.2%)	5 (7.6%)	
	Unemployed	16 (27.1%)	11 (16.7%)	
	Unpaid/voluntary work	1 (1.7%)	0 (0%)	
Marital status	Married	3 (5.1%)	7 (10.6%)	0
	Common-law	4 (6.8%)	7 (10.6%)	
	Divorced/separated	32 (54.2%)	27 (40.9%)	
	Widowed	1 (1.7%)	2 (3.0%)	
	Single/never married	19 (32.2%)	23 (34.8%)	

Numbers indicate mean (SD) for continuous variables and N (%) for categorical variables

In both arms, participants were eligible for a mean of 8.6 prevention and screening actions at baseline (Table 2). At follow-up, participants in the immediate intervention arm met 64.5% of their actions for which they were eligible at baseline versus 42.1% for those in the wait-list arm. The crude rate ratio for immediate arm versus wait-list arm was 1.53 [95% confidence interval 1.29–1.81] and the mixed- Poisson regression model provided a significant estimated rate ratio of 1.53 [95% confidence interval 1.22–1.84]. When adjusting for household income, the rate ratio minimally changed to 1.54 [95% CI 1.24–1.85].

**Table 2:** Eligible actions, met actions, and composite index score for each study arm

	Overall	Immediate	Wait-list
n	125	59	66
Number of eligible actions at baseline, mean (SD)	8.6 (2.5)	8.6 (2.9)	8.6 (2.2)
Number of met actions at follow-up, mean (SD)	4.3 (2.5)	5.2 (2.7)	3.4 (1.9)
(# met/# eligible)*100% (SD)	52.7% (27.9%)	64.5% (27.5%)	42.1% (23.8%)
Median rate [IQR]	55.6% [30.0–71.4%%]	66.7% [52.3–87.5%]	41.4% [25.0–61.9%]

Table 3 presents achievement of the individual actions in the composite index by study arm and by sex for those who were eligible for the action at baseline. The largest improvement between intervention and wait-list arms for both men and women was seen for measurement of waist circumference (71.9% absolute difference overall between study arms: 54.2% for men, 82.4% for women), screening for BMI (72.7% absolute difference overall between study arms: 80.0% for men, 66.7% for women), and breast cancer screening (50.0% absolute difference overall between study arms). Participants in the wait list arm, who had a higher BMI at baseline, performed slightly better than the intervention arm for weight control (for men), physical activity, and healthy diet score. Of note, achievement of these three actions was high in both arms, with a peak of 96.8% of women (30/31 participants) improving their diet score in the wait list group.

**Table 3:** Met actions at 6 months of eligible actions at baseline, overall and by sex for the 66 people in the wait-list arm and 59 people in the intervention arm

Action	Overall			Male			Female		
	**INTERVENTION**	**WAIT LIST**	**Absolute Difference**	**INTERVENTION**	**WAIT LIST**	**Absolute Difference**	**INTERVENTION**	**WAIT LIST**	**Absolute Difference**
FBS/A1C SCREEN	9/31 (29.0%)	5/20 (25.0%)	4.0%	5/11 (45.5%)	1/9 (11.0%)	3.4%	4/20 (20%)	4/11 (36.4%)	−16.4%
BP SCREEN	9/11 (81.8%)	5/12 (41.7%)	40.2%	2/3 (66.7%)	1/6 (16.7%)	50.0%	6/8 (87.5%)	4/6 (66.7%)	20.8%
BP MONITOR	2/3 (66.7%)	2/3 (66.7%)	0%	0/1 (0%)	2/3 (66.7%)	−66.7%	2/2 (100%)	0/0	–
LDL MEASURED	6/19 (31.6%)	5/19 (26.3%)	5.3%	5/11 (45.5%)	3/12 (25.0%)	20.5%	1/8 (12.5%)	2/7 (28.6%)	−16.1%
BREAST SCREENING	4/8 (50.0%)	0/5 (0%)	50.0%	–	–	–	4/8 (50.0%)	0/5 (0%)	50.0%
COLORECTAL SCREENING	1/7 (14.3%)	0/2 (0%)	14.3%	1/3 (33.3%)	0/1 (9%)	33.3%	0/4 (0%)	0/1 (0%)	0%
PAP SCREENING	2/8 (25.0%)	1/9 (11.1%)	13.9%	–	–	–	2/8 (25.0%)	1/9 (11.1%)	13.9%
BMI SCREENING	8/11 (72.7%)	0/9 (0%)	72.7%	4/5 (80.0%)	0/6 (0%)	80.0%	4/6 (66.7%)	0/3 (0%)	66.7%
WAIST CIRCUMFERENCE	39/53 (73.6%)	1/59 (1.6%)	71.9%	11/19 (57.9%)	1/27 (3.7%)	54.2%	28/34 (82.4%)	0/32 (0%)	82.4%
WEIGHT CONTROL	21/39 (53.8%)	28/49 (57.1%)	−3.3%	6/13 (46.2%)	12/25 (48.0%)	−1.8%	15/26 (57.7%)	16/24 (66.7%)	−9.0%
REFERRAL FOR BMI > =25	30/39 (76.9%)	23/49 (46.9%)	30.0%	8/13 (61.5%)	11/25 (44.0%)	17.5%	22/26 (84.6%)	12/24 (50.0%)	34.6%
SMOKING CESSATION	3/27 (11.1%)	1/28 (3.6%)	7.5%	1/14 (7.1%)	0/13 (0%)	7.1%	2/13 (15.4%)	1/15 (6. 7%)	8.7%
CESSATION REFERRAL	16/27 (59.3%)	928 (32.1%)	27.1%	9/14 (64.3%)	3/13 (23.1%)	41.2%	7/13 (53.8%)	6/15 (40.0%)	13.8%
ALCOHOL CONTROL	10/15 (66.7%)	11/21 (52.4%)	14.3%	5/8 (62.5%)	4/10 (40.0%)	22.5%	5/7 (71.4%)	7/11 (63.6%)	7.8%
ALCOHOL REFERRAL	6/15 (40.0%)	2/21 (9.5%)	30.5%	2/8 (25.0%)	1/10 (10.0%)	15.0%	4/7 (57.1%)	1/11 (9.1%)	48.1%
PHYSICAL ACTIVITY IMPROVED	28/45 (62.2%)	36/56 (64.3%)	−2.1%	5/14 (35.7%)	16/26 (61.5%)	−25.8%	23/31 (74.2%)	20/30 (66.7%)	7.5%
ACTIVITY REFERRAL	32/45 (71.1%)	23/56 (41.1%)	30.0%	8/14 (57.1%)	9/26 (34.6%)	22.5%	24/31 (77.4%)	14/30 (46.7%)	30.7%
HEALTHY DIET SCORE	47/52 (90.4%)	56/61 (91.8%)	−1.4%	17/21 (81.0%)	26/31 (83.9%)	−2.9%	30/31 (96.8%)	30/30 (100%)	−3.2%
NUTRITION REFERRAL	35/52 (67.3%)	18/61 29.5%)	37.8%	13/21 (61.9%)	8/31 (25.8%)	36.1%	22/31 (71.0%)	10/30 (33.3%)	37.6%

## Discussion

In this cluster-randomized trial that assessed the effectiveness of the BETTER HEALTH intervention, we found that participants who lived in low-income neighbourhoods and who volunteered to participate in the trial randomized to a visit with a public health nurse in the role of prevention practitioner achieved significantly more (64.5% vs. 42.1%) of the chronic disease prevention and screening actions at six months for which they were eligible. The most notable differences between the two arms were seen for screening for BMI, measuring waist circumference, and breast cancer screening, but participants in the immediate intervention arm performed better on all actions, with the exceptions of improvement of physical activity, weight control and healthy diet score. For these latter three behavioural actions, both arms performed quite well, suggesting that the baseline survey administered by the research coordinator (along with the provided educational materials from the public health unit) may have been a co-intervention, perhaps motivating all participants to make improvements in their own health. Considering that participants in both arms were overweight on average, improvement in these actions relevant to weight may be a particular success of BETTER HEALTH.

In the original BETTER trial, participants were eligible for a mean of 9.0 of 28 (32%) evidence-based screening and prevention actions at baseline [6]. In the current study, participants were eligible for a mean of 8.6 of 19 (45.2%) evidence-based actions. This higher proportion of unmet actions at baseline is not surprising considering that our participants were living with significant socioeconomic disadvantage; many were unemployed, living with low household income and had relatively low education levels. As previously noted, people living with low socioeconomic status are less likely to be up to date on screening and more likely to have cardiovascular risk factors [1, 2, 20–24]. Despite the socioeconomic disadvantage experienced by our study population, we found that the BETTER HEALTH intervention was effective in improving uptake of prevention and screening actions and that retention in the study was quite high (114/126, 90.5%), which likely reflects a high degree of participant support and engagement. The BETTER intervention has also been shown to be successful in both urban and rural primary care settings [6, 25]. Of note, when BETTER was implemented in rural primary care settings, people with lower income improved on the composite index to a greater degree than their counterparts in an adjusted analysis [25]. Taken together, these findings suggest that the BETTER intervention can be effective in a broad variety of settings and for a diverse array of participants who are motivated to make improvements in their health, including in both the primary care and public health contexts, and perhaps especially for those living with social disadvantage.

Existing literature on interventions to improve prevention, screening and chronic disease management among people living with poverty has shown success for patient navigators trained to help address patient-identified screening barriers [26] and address the SDOH that impact screening uptake [27] and for health coaches trained to support participants to accomplish short-term self-identified health and wellness goals [28, 29]. Qualitative research as a part of BETTER HEALTH will explore which components of the intervention led to its success from both the participant and prevention practitioner perspectives and how to make BETTER HEALTH sustainable, and future research will adapt, and explore the effectiveness of, the BETTER intervention for adults under 40 years of age living with social disadvantage, for whom the potential to reduce premature morbidity and mortality is substantial [24, 30]. Importantly, this future research will explore the potential of the adapted intervention in both the primary care and public health contexts, as we recognize that public health resources can be quite limited, particularly considering recent demands created by the COVID-19 pandemic.

This study has several limitations. First, as noted, our research coordinator may have served as a co-intervention. However, this would bias our findings toward the null hypothesis, and the differences we observed might have been even larger if the survey had been self-administered. It is important to note that many participants had low literacy and a sustainable BETTER HEALTH intervention would likely require an interviewer-administered health survey. Second, we did not engage with the primary care providers of study participants and only one participant asked to be connected to the primary care provider arranged by the study team. Connection of the prevention practitioners with participants’ primary care providers may have led to greater improvement in actions that required a provider’s involvement, such as cancer screening and lipid and glucose testing, in the intervention arm. Third, we relied exclusively on self-report from study participants, which is subject to recall and social desirability bias (the tendency to under report socially undesirable actions and to over report socially desirable actions) [31]. However, we wanted to ensure that our intervention would be effective for those with no, or loose, primary care contact, and thus did not use medical record data. Fourth, we excluded non-English speakers, excluding a sociodemographic group that is likely at even higher risk of inadequate medical care. Fifth, we did not explore the role of other sociodemographic characteristics such as race/ethnicity and immigration status that may intersect with income. Sixth, we do not know if changes documented at six months, such as improvement in diet and physical activity, were sustained beyond that time period. Health-related behaviour change is notoriously hard to maintain in the long term [32–34]. Finally, we did not explicitly compare the clusters based on factors that may have affected uptake of recommendations, such as proximity to primary care or access to public transportation. However, we received input from public health on our clusters who felt that the included clusters were comparable.

## Conclusion

Through a wait list-control cluster-randomized trial, BETTER HEALTH, we showed that public health nurses in the role of prevention practitioners using the BETTER intervention were effective in increasing the proportion of identified evidence-based prevention and screening actions achieved at six months for people living with socioeconomic disadvantage who are motivated to make improvements in their health. Policymakers interested in primary and secondary prevention of chronic diseases and cancers should consider incorporating this approach into their jurisdictional contexts.

## Data Availability

The datasets generated during and/or analysed during the current study are not publicly available due to lack of consent from participants or approval from our ethics boards to do to.

## Appendix

**Table 4:** Details of actions included in BETTER HEALTH: Durham study outcome composite index. Adapted from composite index used in original BETTER trial [6, 8]

		Eligible for:	Met if:
1	FBS or A1C screen	A) All non-diabetics with at least one risk factor or self-report as pre-diabetic that haven’t had a Fasting Blood Sugar (FBS) or A1C in the past year OR B) All non-diabetics without risk factors (see Section B) that haven’t had an FBS or A1C in the past 3 years OR C) All non-diabetics with pre-diabetes who haven’t had a FBS/A1C in past 6 months	Had a fasting blood test to check for diabetes or had an A1C test since the first survey interview
2	BP screen	A) All patients without CVD and Non-hypertensive and Non-diabetic > 12 months since BP check OR B) All patients without CVD and Non-hypertensive and Diabetic > 6 months since BP check	Had a nurse or doctor check blood pressure since the first survey interview
3	BP monitor	All patients without CVD with hypertension with no BP check in past 6 months	Had a nurse or doctor check blood pressure since the first survey interview
4	LDL measured	A) All non-diabetic Men ≥ 40 without CVD without a cholesterol test reported < 1 year ago OR B) All non-diabetic Women ≥ 50 without CVD without a cholesterol test reported < 1 year ago	Had a fasting blood test to check for high cholesterol since the first survey interview
5	Breast screening	A) All women ≥ 50-65 without a personal history of breast cancer AND without family history risk of breast or ovarian cancer: Routine mammogram not done within 2 years OR OR B) All women 40-65 without personal history of breast cancer but with a family history risk of breast or ovarian cancer: Routine mammogram not done within 1 year	Had a mammogram since the first survey interview
6	Colorectal screening	A) All patients ≥50-65 without personal history CRC and without family history of CRC with the following: ◦ FOBT not done within 2 years ◦ Sigmoidoscopy not done within 5 years ◦ Colonoscopy not done within 10 years OR B) All patients 40-65 with a family history of CRC with the following: ◦ FOBT not done within 2 years ◦ Sigmoidoscopy not done within 5 years ◦ Colonoscopy not done within 10 years	Had an FOBT or colonoscopy or sigmoidoscopy since the first survey interview
7	Pap screening	All women without personal history of cervical cancer with no pap tests within the past 3 years	Had a Pap test since the first survey interview
8	BMI screening	BMI not measured in past 2 years	Had a nurse or doctor measure height and weight since the first survey interview
9	Waist circumference	Waist circumference not measured in past 2 years	Had a nurse or doctor measure waist circumference since the first survey interview
10	Weight Control	BMI ≥ 25	No increase in BMI since the first survey interview
11	Referral for BMI >= 25	BMI ≥ 25	Been referred to an exercise program, been referred to a dietician/nutrition program, had a discussion with a health professional about exercise, had a discussion with a health professional about diet/nutrition, initiated a referral to a diet/nutrition program, or initiated a referral to an exercise program since the first survey interview
12	Smoking cessation	Smoker	Not smoking cigarettes at all at time of 6-month survey interview
13	Cessation referral	Smoker	Prescribed medication for smoking cessation, been referred to a smoking cessation program, had a discussion with a health professional about smoking cessation, or initiated a referral to a smoking cessation program since the first survey interview
14	Alcohol control	A) ≥ 7 drinks per week for Women or AUDIT-C (Alcohol Use Disorders Identification Test) [35] score >3 OR B) ≥ 14 drinks per week for Men or AUDIT-C score ≥ 4	AUDIT-C score from 6-month survey interview less than on first survey interview, or number of drinks per week at 6-moth survey interview less than on first survey interview
15	Alcohol referral	A) ≥ 14 drinks per week or AUDIT-C score >4 for Men OR B) ≥ 7 drinks per week or AUDIT-C score >3 for Women* OR C) Identified as a binge drinker (>6 drinks on one occasion)	Prescribed medication for alcohol cessation, been referred to an alcohol cessation program, had a discussion with a health professional about alcohol cessation, or initiated a referral to an alcohol cessation program since the first survey interview
16	Physical activity improved	< 150 minutes of vigorous physical activity per week OR General Practice Physical Activity Questionnaire (GPPAQ) score is other than “active” [36]	Increase in the number of minutes spent exercising per week since the first survey interview or GPPAQ score improved on 6-month survey interview compared to first survey interview
17	Activity referral	< 150 minutes of vigorous physical activity per week OR General Practice Physical Activity Questionnaire (GPPAQ) score is other than “active”	Been referred to an exercise program, had a discussion with a health professional about exercise, or initiated a referral to an exercise program since the first survey interview
18	Healthy diet score	Starting the Conversation diet score ≥ 4	Decrease in diet score on 6-month survey interview from first survey interview
19	Nutrition referral	Starting the Conversation diet score ≥ 4 [37]	Been referred to a dietician/nutrition program, had a discussion with a health professional about diet/nutrition, or initiated a referral to a diet/nutrition program since the first survey interview

## Abbreviations

BETTER HEALTH: Building on Existing Tools to Improve Chronic Disease Prevention and Screening in Public Health
SDOH: social determinants of health
