# Correction to: Building on existing tools to improve chronic disease prevention and screening in public health: a cluster randomized trial

**DOI:** 10.1186/s12889-021-11700-0

**Published:** 2021-09-21

**Authors:** A. K. Lofters, M. A. O’Brien, R. Sutradhar, A. D. Pinto, N. N. Baxter, P. Donnelly, R. Elliott, R. H. Glazier, J. Huizinga, R. Kyle, D. Manca, M. A. Pietrusiak, L. Rabeneck, B. Riordan, P. Selby, K. Sivayoganathan, C. Snider, N. Sopcak, K. Thorpe, J. Tinmouth, B. Wall, F. Zuo, E. Grunfeld, L. Paszat

**Affiliations:** 1 Department of Family & Community Medicine, University of Toronto, Toronto, Canada; 2 Women’s College Hospital Research Institute, Toronto, Canada; 3 Peter Gilgan Centre for Women’s Cancers, Women’s College Hospital, Toronto, Canada; 4 Ontario Health (Cancer Care Ontario), Toronto, Canada; 5 ICES, Toronto, Canada; 6 Institute of Health Policy, Management and Evaluation, University of Toronto, Toronto, Ontario Canada; 7 MAP Centre for Urban Health Solutions, St. Michael’s Hospital, Toronto, Canada; 8 Department of Family and Community Medicine, St. Michael’s Hospital, Toronto, Canada; 9 Division of Biostatistics, Dalla Lana School of Public Health, University of Toronto, Toronto, Ontario Canada; 10 Dalla Lana School of Public Health, University of Toronto, Toronto, Canada; 11 University of St. Andrews, Scotland, UK; 12 Durham Region Health Department, Whitby, Canada; 13 Melbourne School of Global and Population Health, University of Melbourne, Melbourne, Australia; 14 Department of Family Medicine, University of Alberta, Edmonton, Canada; 15 Centre for Addiction and Mental Health, Toronto, Canada; 16 Applied Health Research Centre, St. Michael’s Hospital, Toronto, Canada; 17 Sunnybrook Health Sciences Centre, Toronto, Canada; 18 Ontario Institute for Cancer Research, Toronto, Canada

**Correction to: BMC Public Health 21, 1496 (2021)**

**https://doi.org/10.1186/s12889-021-11452-x**

It was highlighted that the original article [1] contained some mistakes in some of the authors’ affiliations. This Correction article shows the correct affiliations. The original article has been updated.
